# Mitonuclear and phenotypic discordance in an Atlantic Forest frog hybrid zone

**DOI:** 10.1002/ece3.70262

**Published:** 2024-09-13

**Authors:** Fábio P. de Sá, Maria Akopyan, Erika M. Santana, Célio F. B. Haddad, Kelly R. Zamudio

**Affiliations:** ^1^ Departamento de Biodiversidade and Centro de Aquicultura (CAUNESP) Instituto de Biociências, Universidade Estadual Paulista (UNESP) Rio Claro São Paulo Brazil; ^2^ Department of Ecology and Evolutionary Biology Cornell University Ithaca New York USA; ^3^ Departamento de Ecologia Instituto de Biociências, Universidade de São Paulo (USP) São Paulo Brazil; ^4^ Department of Integrative Biology University of Texas at Austin Austin Texas USA

**Keywords:** anuran, body size, intrasexual competition, introgression, mitonuclear discordance, sexual selection

## Abstract

Discordance between mitochondrial and nuclear DNA is common among animals and can be the result of a number of evolutionary processes, including incomplete lineage sorting and introgression. Particularly relevant in contact zones, mitonuclear discordance is expected because the mitochondrial genome is haploid and primarily uniparentally inherited, whereas nuclear loci are evolving at slower rates. In addition, when closely related taxa come together in hybrid zones, the distribution of diagnostic phenotypic characters and their concordance with the mitochondrial or nuclear lineages can also inform on historical and ongoing dynamics within hybrid zones. Overall, genetic and phenotypic discordances provide evidence for evolutionary divergence and processes that maintain boundaries among sister species or lineages. In this study, we characterized patterns of genetic and phenotypic variation in a contact zone between *Cycloramphus dubius* and *Cycloramphus boraceiensis*, two sister species of frogs endemic to the Atlantic Coastal Forest of Brazil. We examined genomic‐scale nuclear diversification across 19 populations, encompassing the two parental forms and a contact zone between them. We compared the distribution of genomic DNA variability with that of a mitochondrial locus (16S) and two morphological traits (dorsal tubercles and body size). Our results reveal multiple divergent lineages with ongoing admixture. We detected discordance in patterns of introgression across the three data types. *Cycloramphus dubius* males are significantly larger than *C. boraceiensis* males, and we posit that competition among males in the hybrid zone, coupled with mate choice by females, may be one mechanism leading to patterns of introgression observed between the species.

## Graphical abstract

We characterized the patterns of genetic and phenotypic variation in a contact zone between *Cycloramphus dubius* and *Cycloramphus boraceiensis*, two sister species of frogs endemic to the Atlantic Coastal Forest of Brazil. We detected discordance in patterns of introgression across the three data types. *Cycloramphus dubius* males are significantly larger than *C. boraceiensis* males, and we posit that competition among males in the hybrid zone, coupled with mate choice by females may be one mechanism leading to patterns of introgression observed between the species. Photo credit: Leo R. Malagoli.

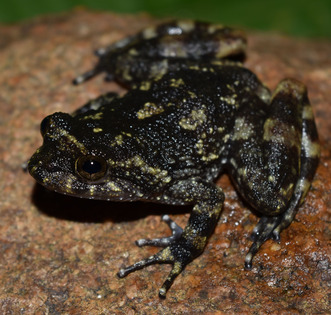

## 1 INTRODUCTION

Animal species often show intraspecific geographic variation associated with diversification (Coyne & Orr, 2004; Endler, 1977; Lee et al., 2019). Even in recently diverged species, microhabitat or topographic variation can isolate lineages, facilitating diversification and speciation (Devitt et al., 2011; Sánchez‐Montes et al., 2018). The divergence of populations can lead to premating barriers that prevent interbreeding when these populations are again in sympatry (Pfennig & Rice, 2014; Seddon, 2005). However, these premating isolating barriers are often imperfect and divergent species that subsequently come into secondary contact often interbreed, resulting in a range of possible outcomes (e.g., Maag et al., 2023; Souza et al., 2024). In cases where hybrid offspring occur in zones of secondary contact, postmating barriers may evolve, reinforcing the distinction between species through unfit hybrids, a consequence of parental genomic incompatibility or reduced ecological competitive fitness (Coyne & Orr, 2004; Singhal & Moritz, 2012; Yoshida et al., 2019). Conversely, hybridization and the subsequent backcrossing can promote introgressive hybridization, a process known across a wide range of animal taxa, providing a source of novel and potentially adaptive genetic variation in hybrid populations (Anderson, 1948; Dufresnes & Martínez‐Solano, 2020; Grant & Grant, 1994; Lewontin & Birch, 1966; Todesco et al., 2016). Clearly, the outcome of interactions between species across secondary contact zones is highly variable and complex, and understanding these variable outcomes informs on mechanisms leading to the maintenance of divergent lineages or incipient species.

Populations experiencing secondary contact often show discordant patterns of admixture (Devitt et al., 2011; Johnson et al., 2015; Lipshutz et al., 2019; Pfennig & Simovich, 2002; Veen et al., 2001), which result from pre‐ and post‐zygotic isolating mechanisms (Moran et al., 2019; Sasa et al., 1998; Veen et al., 2001; Wirtz, 1999). For genetic introgression, distinct selective constraints, such as coding or noncoding loci, effective population sizes, and whether introgression occurs in organellar or autosomal DNA, can lead to genealogical discordances (Maddison, 1997; Singhal & Moritz, 2012). Discordance between organellar and nuclear loci has been identified in many taxa, particularly in contact zones (Edwards, 2009; Toews & Brelsford, 2012). These discordances are expected because the mitochondrial genome is haploid and typically uniparentally inherited, whereas nuclear loci and phenotypic traits are presumably evolving at slower rates. In some cases, regions of discordance in mitochondrial and nuclear genetic patterns are also coincident with variability in phenotypes that mediate mate selection (Harris et al., 2018; Toews & Brelsford, 2012; Wham et al., 2021). Thus, studies of contact zones have the potential to reveal selective, behavioral, and demographic mechanisms acting on populations, such as differential selection (Cheviron & Brumfield, 2009), sex‐biased dispersal (Crochet et al., 2003), mechanisms underlying mate choice (Wirtz, 1999), historical spatial expansion or contraction, and the genetic drift (Currat et al., 2008).

Among vertebrates, amphibians are highly phylopatric and often have specific microhabitat preferences (Wells, 2010; Zeisset & Beebee, 2008). In the Atlantic Forest, frogs are highly diverse and have high levels of endemism due to specialized microhabitat use (Heyer & Maxson, 1983; Silva et al., 2012; Vasconcelos et al., 2019). Nonetheless, hybridization between anuran sister species does occur (Haddad et al., 1994). The high diversity in the Atlantic Coastal Forest biome stems from historically dynamic shifts in habitat distributions that resulted in multiple contractions and expansions of species ranges, promoting opportunities for lineage divergences and hybridization among differentiated forms (Amaral et al., 2018; Carnaval et al., 2014; Thomé et al., 2014). Here, we characterized patterns of genetic and phenotypic variation along a contact zone between *Cycloramphus dubius* and *C. boraceiensis*, two sister species of frogs endemic to the Atlantic Coastal Forest in southeastern Brazil that have largely parapatric distributions (de Sá, Haddad et al., 2020; Haddad et al., 2013). Our focal species occur in the states of São Paulo and Rio de Janeiro, and have highly similar ecological niches; both are saxicolous, living and breeding in crevices formed by rocks or roots at the margins of fast‐flowing streams in forested environments, commonly next to waterfalls (Giaretta & Cardoso, 1995; Giaretta & Facure, 2003; Haddad et al., 2013; Haddad & Prado, 2005; Pedrozo et al., 2024).

Among frogs, females typically choose mates based on territorial and/or phenotypic traits, such as breeding site and/or male body size, therefore enhancing competition among males (de Sá, Consolmagno et al., 2020; Muralidhar et al., 2014; O'Brien et al., 2020; Richardson et al., 2010; Roithmair, 1994). In our two focal species of *Cycloramphus*, males actively compete and defend territories, where they mate and guard their egg clutches after reproduction, setting the stage for female choice based on these behaviors (de Sá, Haddad et al., 2020; Giaretta & Cardoso, 1995; Giaretta & Facure, 2003; Haddad & Prado, 2005). *Cycloramphus dubius* and *C. boraceiensis* are morphologically similar, but diagnosable because *C. dubius* lacks distinct dorsal white tubercles that are present in *C. boraceiensis* (Heyer, 1983). Furthermore, both species have similar karyotypes (2*n* = 26; Beçak et al., 1970; Noleto et al., 2011; Silva et al., 2001) and have partially sympatric distributions, thus providing a good case for the study of gene flow and potential introgression between recently diverged species.

We explore divergence between our two focal frog species with reduced representation genomic data (nuDNA) and compare genomic variation to the distribution of mitochondrial variation (mtDNA), dorsal morphology, and body size along a secondary contact zone. Our goals in this study are to: (1) characterize the contact zone and the outcome of gene flow between our two focal species; and (2) explore potential evolutionary mechanisms that might have governed introgression in different character types. By assessing how secondary contact affects the distribution of genetic and phenotypic diversity, our study advances the understanding of diversification and speciation processes in the Atlantic Forest, a biodiversity hotspot (Marques & Grelle, 2021; Morellato & Haddad, 2000; Myers et al., 2000; Vasconcelos et al., 2014).

## 2 MATERIALS AND METHODS

### 2.1 Sample collection

**FIGURE 1:**
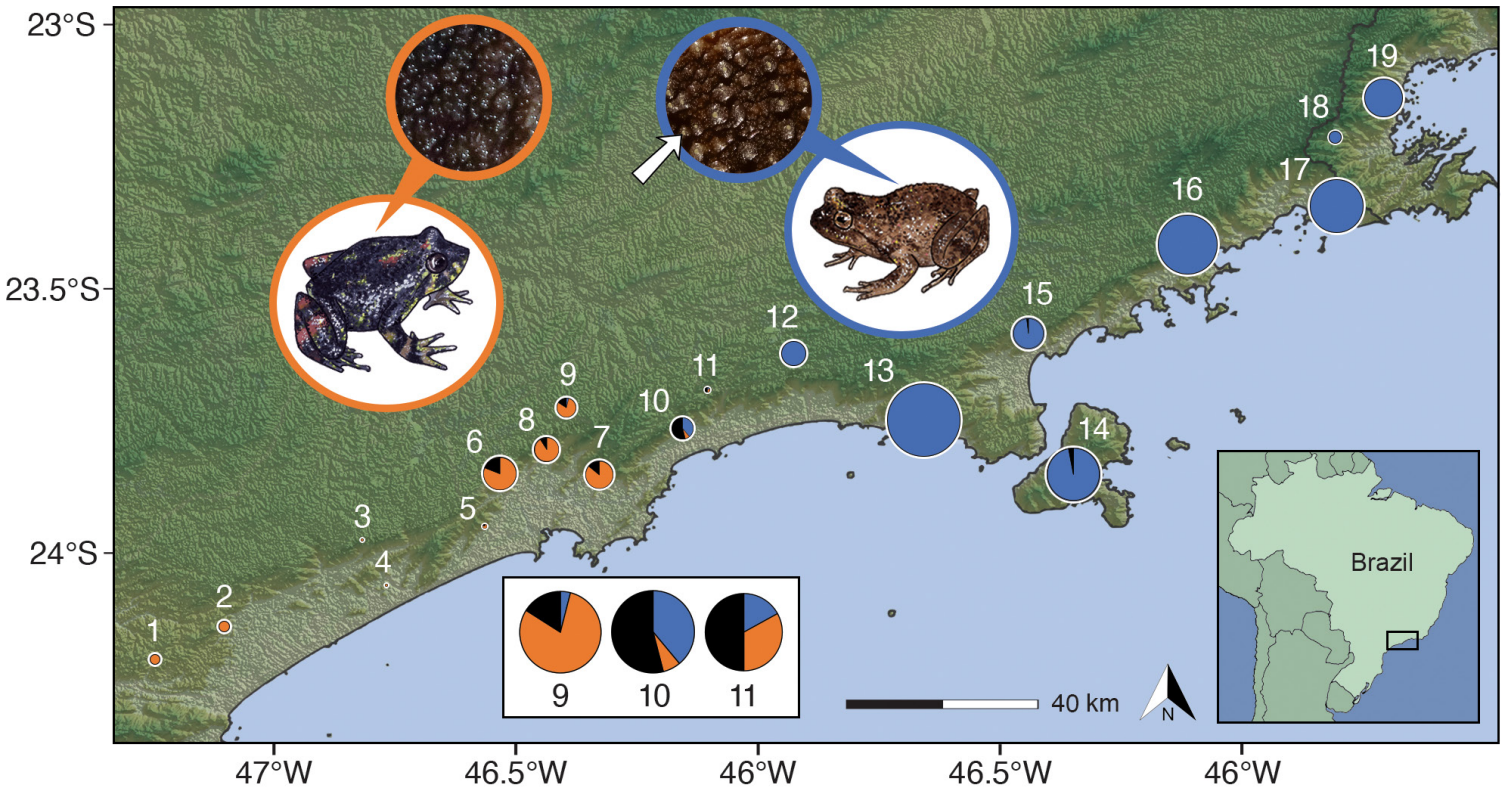
Population sampling of *Cycloramphus dubius* and *Cycloramphus boraceiensis* in the states of São Paulo and Rio de Janeiro, southeastern Brazil. Based on mtDNA and nuDNA, *C. dubius* occurs at sites 1–11, lineage *C. boraceiensis* L1 at sites 12–13, insular *C. boraceiensis* population at site 14, and *C. boraceiensis* L2 at sites 15–19. Numbered populations are defined in Table 1. Images are representatives of each species, which are distinguished based on dorsal morphology (Heyer, 1983). *Cycloramphus dubius* lacks distinct dorsal white tubercles that are present in *C. boraceiensis* (white arrow points to a white tubercle). Numbered circles represent sampled populations and are color‐coded according to the proportions of the three dorsal morphologies: Orange represents the morphology typical of *C. dubius*, blue represents the morphology typical of *C. boraceiensis*, and black represents the intermediate morphology. Numbered circles are sized according to the sample sizes of specimens examined for dorsal morphology (Table 1). Populations 9, 10, and 11 exhibit the three dorsal morphologies, so their circles are enlarged inside the white box for easier visualization.

*Cycloramphus dubius* and *C. boraceiensis* are saxicolous frogs that are largely parapatric and their ranges come into contact along the central coast of the São Paulo state, near the municipalities of Biritiba Mirim and Salesópolis (de Sá, Haddad et al., 2020). From 2011 to 2016, we sampled populations of both species across their geographic distributions, collecting frogs and tadpoles in the field by hand at night. We supplemented those with tissue samples from previous collections from our laboratories, for a total of 302 individuals from 19 populations. We euthanized field collected frogs and tadpoles with anesthetic overdose (5% lidocaine) and preserved tissues from tadpoles (fins or the whole specimens), juveniles, and adults (livers, toe tips, or thigh muscles) in 100% ethanol. All vouchers and tissues are deposited in the Célio F. B. Haddad amphibian collection (CFBH), Departamento de Biodiversidade, Instituto de Biociências, Universidade Estadual Paulista (UNESP), Rio Claro, São Paulo, Brazil. For these same populations, we collected data on species‐level diagnostic dorsal morphologies (for juveniles and adult females and males; described in phenotypic data section below) and body size (for adult females and males). We supplemented phenotypic data from our own field samples with data from museum collections, resulting in data for 721 individuals (Table 1 and Figure 1). Dorsal morphology was characterized for 653 specimens and body size measurements were taken from 371 adults (Table 1). Focusing on the potential contact zone, we sampled outward from there to include most of the areas in which *C. dubius* and *C. boraceiensis* occur, only lacking frogs from the extreme northeastern range of *C. boraceiensis* (in the municipality of Angra dos Reis; Bittencourt‐Silva & Silva, 2013). We included samples from the type localities of both species: Alto de Paranapiacaba (site 9, ALTO; Table 1), type locality of *C. dubius*; and Salesópolis (site 12, SALE; Table 1), type locality of *C. boraceiensis*.

**TABLE 1:** Population sampling of *Cycloramphus dubius* (sites 1–11) and *Cycloramphus boraceiensis* (sites 12–19) from southeastern Brazil.

Site	Acronym	Population	16S (*n*)	SNP (*n*)	Dorsal morphology (*n*)	Body size (*n*)	Geographical coordinates
1	PTOL	Pedro de Toledo, SP	10	10	13	7	24°15'4" S, 47°13'28" W
2	PERU	Peruíbe, SP	16	16	14	9	24°10'53" S, 47°0'42" W
3	ITAN	Itanhaém, SP	6	6	1	1	23°59'49" S, 46°44'30" W
4	MONG	Mongaguá, SP	0	0	1	1	24°5'46" S, 46°40'59" W
5	SVIC	São Vicente, SP	2	2	5	4	23°56'46" S, 46°29'28" W
6	SPSA	Road São Paulo‐Santos, SP	1	1	43	28	23°51'52" S, 46°27'44" W
7	SANT	Santos, SP	32	22	36	29	23°52'46" S, 46°16'35" W
8	CUBA	Cubatão, SP	25	20	32	25	23°48'44" S, 46°21'17" W
9	ALTO	Alto de Paranapiacaba, SP	0	0	25	12	23°46'48" S, 46°17'54" W
10	BERT	Bertioga, SP	17	15	28	19	23°46'39" S, 46°7'3" W
11	BIRI	Biritiba Mirim, SP	2	1	6	2	23°42'29" S, 46°2'54" W
12	SALE	Salesópolis, SP	2	2	32	4	23°38'10" S, 45°56'38" W
13	SSEB	São Sebastião, SP	78	69	96	48	23°42'58" S, 45°45'17" W
14	IBEL	Ilhabela, SP	36	35	70	49	23°52'30" S, 45°25'36" W
15	CARA	Caraguatatuba, SP	25	25	41	18	23°35'10" S, 45°25'5" W
16	UBAT	Southwestern Ubatuba, SP	2	2	78	50	23°24'57" S, 45°6'59" W
17	PICI	Picinguaba, Northeastern Ubatuba, SP	28	27	72	36	23°21'53" S, 44°47'29" W
18	PACU	Road Paraty‐Cunha, RJ	5	5	17	7	23°12'28" S, 44°49'27" W
19	PARA	Paraty, RJ	15	15	43	21	23°5'11" S, 44°43'30" W

*Note*: We report the locality (population) and final sample sizes for genetic and phenotypic data included in downstream analyses. Populations are numbered according to the map in Figure 1.

Abbreviations: RJ, Rio de Janeiro state; SP, São Paulo state.

### 2.2 Mitochondrial DNA sequencing and analysis

We isolated total genomic DNA from tissues using a DNeasy Blood & Tissue Kit (Qiagen) following the manufacturer's protocol. Using polymerase chain reaction (PCR), we amplified and sequenced the mitochondrial 16S ribosomal DNA gene fragment (mtDNA, 16S; 615 base pairs, bps; Palumbi et al., 1991) using primers 16Sa‐L (5′‐CGCCTGTTTATCAAAAACAT‐3′) and 16Sb‐H (5′‐CCGGTCTGAACTCAGATCACGT‐3′). We carried out PCRs in a final volume of 25 μL, each containing 1–10 ng DNA template, 1X PCR Buffer (with 1.5–3 mM MgCl_2_), 1X bovine serum albumin (BSA), 0.4 μM of forward and reverse primers, 0.76 mM dNTPs, and 0.625 units of Taq polymerase (Roche). Amplification conditions included an initial denaturation at 94°C for 5 min, followed by 35–40 cycles of denaturation at 94°C for 1 min, annealing at 53°C for 1 min, extension at 72°C for 1 min, followed by a final extension at 72°C for 5–10 min. We purified final PCR products using 1X SAP buffer, SAP enzyme (1 unit; Life Technologies), and exonuclease I (10 units; Life Technologies). We sequenced purified PCR products in both directions using BigDye terminator sequencing chemistry (Applied Biosystems) on an ABI automated 3730xl Genetic Analyzer (Applied Biosystems), and obtained good quality mitochondrial sequences for a total of 302 individuals (Table 1).

We used the program TCS v. 1.21 (Clement et al., 2000) to generate a haplotype network. The connection limit was set at 95% and gaps in sequences were read as missing data. The haplotype network was visualized using tcsBU (dos Santos et al., 2016).

### 2.3 Genomic DNA sequencing

Following methods in Peterson et al. (2012), we generated double digest restriction‐site associated DNA sequence libraries (ddRADseq) and collected genome‐wide single nucleotide polymorphism (SNP) data to characterize nuclear genetic diversification along our hybrid transect (nuDNA). We included uniquely barcoded/indexed individuals in six libraries (three libraries with 40 individuals each and another three with 56 individuals each), including all those samples for which we had appropriate concentration of DNA. We combined digestion and adapter ligation steps, using 100 ng genomic DNA (10.5 μL at 10 ng μL−1), 15 units of both SbfI‐HF and MspI (NEB; 0.75 μL each), 300 units of T4 DNA ligase (NEB, 0.75 μL), 3 μL of 10 × CutSmart ligase buffer (NEB), 1.0 μL each of a uniquely barcoded SbfI‐P1 adapter (one of eight) and MspI‐P2 adapter (Integrated DNA Technologies; 0.25 μM each), 3 μL of 10 mM ATP (NEB), and 9.25 μL of ddH2O for a total reaction volume of 30 μL. We incubated the restriction‐ligation mixture at 37°C for 30 min and then at 20°C for 1 h. We pooled 20 μL from each of the eight barcoded samples. We PCR‐amplified each pool using 20 ng P1/P2 adapted DNA template (10 μL at 2 ngμL−1), 12.5 μL 2X Phusion PCR Master Mix (2X, NEB), and 1.25 μL each of forward index primer and a uniquely indexed reverse primer (one of seven; 5 μM each, Integrated DNA Technologies). We pooled PCRs across index groups and size‐selected them to retain fragments from 300 to 1,000 bps. Each library was single‐end sequenced to 100 bps on one lane of the Illumina HI‐SEQ 2500 at the Cornell University Genomics Facility, yielding good quality sequences for 273 individuals (Table 1).

### 2.4 Quality filtering and SNP calling

Reads were processed in STACKS v. 1.48 (Catchen et al., 2013) with the module process_radtags to discard low‐quality reads and reads with ambiguous barcodes or RAD cut sites. The remaining reads were demultiplexed to produce fastq files for each frog individual. Next, we used the module denovo_map to merge stacks (i.e., identical set of reads) into loci within individuals and to build a catalog of loci across individuals. We set the minimum depth of coverage required to create a stack (−m option) and the maximum number of pairwise differences allowed between any two stacks within a locus (−M option) to three reads. We allowed a maximum of one mismatch when building the catalog of loci across individuals (−n option). Variants were called using the default SNP model with a genotype likelihood ratio test critical value (*α*) of 0.05. Using the module populations, we filtered SNPs to retain those present in at least two sampling sites (*p* = 2) and present in 50% of individuals (*r* = 0.5). We further filtered our data to remove sequence reads with low depth of coverage (<2X per individual) and/or a minor allele frequency less than 0.5%.

### 2.5 Population genomic analyses: PCAs, structure, hybrid ancestry, and phylogeny

Using R v. 3.5.2 (R Core Team, 2018), we conducted a principal component analysis (PCA) on the centered, unscaled nuDNA genotype matrix to visualize and infer patterns of population structure first using the full dataset. Our initial analysis of the full dataset showed clearly that individuals from Ilhabela (site 14, IBEL; a continental island surrounded by sea water, a natural barrier for frogs) are highly isolated and form their own genetic deme. Therefore, we excluded this insular population from some of our downstream analyses, to enhance the visualization of admixture patterns among populations sampled on a transect across the ranges and the hybrid zone of *C. dubius* and *C. boraceiensis*. We further quantified population structure and examined nuDNA genetic admixture using Entropy (Gompert et al., 2014), a Bayesian method that deals with uncertainty in nuDNA genotypes and estimates the proportion of ancestry of a set of individuals from contributing populations. Next, we used the adegenet R package v. 2.1.1 to identify the best‐fitting number of clusters given the Bayesian Information Criterion (BIC) values for K ranging from 1 to 12. This optimal clustering solution was then used to perform a discriminant analysis of principal components (DAPCs), a multivariate analysis that minimizes within‐group variance while maximizing among‐group variance (Jombart et al., 2010). Next, we estimated hybrid ancestry between *C. dubius* and two identified lineages of *C. boraceiensis*. To determine the ancestry of the putative hybrids, we assigned two source populations and calculated the fraction of nuDNA loci that combine ancestry from these two parental populations in Entropy (Gompert & Buerkle, 2016). We generated triangle plots (Gompert & Buerkle, 2016), where hybrids with different admixture proportions (*q*) are aligned along the *x*‐axis and ranked according to the fraction of loci at which each individual has ancestry from both parental taxa (Q_12_, interpopulation ancestry, *y*‐axis). This analysis allows us to separate hybrids that are progeny from a cross involving one or both parental taxa (backcrosses or F_1_) and thus have maximal interpopulation ancestry for a given admixture proportion (on the edges of the triangle; Gompert & Buerkle, 2016), from progeny of crosses between hybrid individuals (F_2_ and beyond) that have less than maximal interpopulation ancestry for a given admixture proportion (middle of the triangle; Gompert & Buerkle, 2016). The insular population (site 14, IBEL) was excluded from the hybrid ancestry analysis.

To understand the phylogenetic relationships between *C. dubius* and *C. boraceiensis* populations, we first built a maximum likelihood (ML) phylogeny using RAxML‐NG v. 1.2.2 (Kozlov et al., 2019) in the CIPRES Science Gateway v. 3.3 (Miller et al., 2010). We used the full SNP dataset, including the insular population. The best‐fit nucleotide substitution model for our dataset was determined by the corrected Akaike Information Criterion (AICc) in the jModelTest v. 2.1.8. (Darriba et al., 2012). We applied the GTR + I + G nucleotide substitution model, with 20 starting trees (10 random and 10 parsimony trees), and assessed branch support by running 1000 bootstrap replicates. We used FigTree v. 1.4.4 to edit the final tree (Rambaut, 2018), apllying midpoint rooting.

We also inferred population trees using a coalescent model and Bayesian MCMC approach implemented in SNAPP (Bryant et al., 2012). SNAPP is a tool developed for phylogenetic analyses based on biallelic markers (such as SNPs), directly inferring species trees by integrating over all possible genealogies rather than sampling them. This approach provides high statistical power but is computationally expensive, with complexity scaling according to the number of samples and markers (Yoder et al., 2013). For our analyses, we used SNAPP as implemented in BEAST v. 2.7.6 (Bouckaert et al., 2019) via CIPRES. We used the default model and prior parameters (models: μ = 1, *v* = 1, coalescence rate = 10; priors: *α* = 11.75, *β* = 109.73, *κ* = 1, and λ = 0.01) and ran two independent replicate MCMC runs of 1000,000 iterations with sampling every 1000 steps and a burn‐in of 10%. To ensure feasible computational times, we reduced the SNP dataset to 2408 SNPs, randomly selected from the full dataset (of 5972 SNPs), and included only 12 randomly chosen individuals from each lineage (*C. dubius*, *C. boraceiensis* L1, insular *C. boraceiensis*, and *C. boraceiensis* L2), for a total of 48 individuals (Rodger et al., 2021; Stetter & Schmid, 2017). We used the preliminary ML topology (Figure S1) to guide our sample choice and also included in the reduced dataset those samples that were not grouping with their own populations. This necessarily biases topologies and parameter estimates in our cloudogram but also represents the full discordance among genealogies of hybridizing lineages.

We assessed the output using Tracer v. 1.7.2 (Rambaut et al., 2018) to ensure acceptable mixing and convergence, confirming effective sample sizes >200 for all parameters. The two runs for each replicate were combined using LogCombiner v. 2.7.4 (Drummond & Rambaut, 2007), using a burn‐in of 10%. The posterior distribution of gene (SNP) trees was visualized using DensiTree v. 2.6.1 (also implemented in BEAST; Bouckaert, 2010) to capture uncertainty in topology and branch lengths. Finally, we summarized the data in a maximum clade credibility (MCC) tree using TreeAnnotator v. 2.7.4 (also implemented in BEAST; Drummond & Rambaut, 2007), after discarding the first 20% of samples as burn‐in.

### 2.6 Phenotypic data and analyses

At each sampling site, we collected data on dorsal morphology and adult body size (snout–vent length, SVL). We augmented our phenotypic dataset by scoring dorsal morphologies and measuring sizes of specimens at four institutions in Brazil: CFBH, Rio Claro, São Paulo; Museu de Zoologia da Universidade de São Paulo (MZUSP), São Paulo; Museu Nacional, Universidade Federal do Rio de Janeiro (MNRJ), Rio de Janeiro; and Museu de Diversidade Biológica (MDBio), Universidade Estadual de Campinas (ZUEC), Campinas, São Paulo. We implemented the scoring system proposed by Heyer (1983) to categorize *Cycloramphus* species based on variation in dorsal morphology. We categorized individuals into one of two dorsal morphology types: uniform (free from white tubercles, typical of *C. dubius*) or white‐tubercled (fully covered by white tubercles, typical of *C. boraceiensis*; Figure 1). We also discovered a novel combination of the two basic dorsal morphology types, which we categorized as “intermediate”, in which the dorsum is partially covered by white tubercles. We calculated the proportion of individuals of the three dorsal types at each sampling site. We measured adult SVL with calipers (±0.1 mm). To estimate sexual size dimorphism, we used a size dimorphism index, calculated as the ratio of female to male body size subtracted by one [SDI: (female SVL/male SVL) – 1; Lovich & Gibbons, 1992]. Our data were normally distributed (Shapiro–Wilk test), and showed equality of variances (Levene's test), so we compared averages of female and male body sizes, and sexual size dimorphism among genetic lineages using *t*‐test (*t*) or one‐way ANOVA test (*F*) followed by Tukey test (*Q*'), in R v. 3.5.2 (R Core Team, 2018). We considered statistical significance when *p* < .05 (Zar, 1998).

### 2.7 Geographic cline analyses

To estimate the extent of introgression between *C. dubius* and *C. boraceiensis*, we estimated clinal transitions of mitochondrial haplotype frequencies, allele frequencies and admixture proportions from the SNP dataset, and dorsal morphology frequencies. We fit genetic and phenotypic data to geographic cline models using the Metropolis–Hasting Markov chain Monte Carlo algorithm implemented in the R package hzar (Derryberry et al., 2014). We excluded the insular population (site 14, IBEL) from the geographic cline analyses. To generate clines, we calculated pairwise Euclidean distances (in km) from the southern‐most sampling site from geographic coordinates with the program Geographic Distance Matrix Generator v. 1.2.3 (Ersts, 2011). Mitochondrial haplotype data were coded as 1 or 0, with 1 indicating that the haplotype belonged to the *C. dubius* mitochondrial clade and 0 indicating that the haplotype belonged to one of the two mitochondrial lineages of *C. boraceiensis*. Clinal transitions of admixture proportions from the SNP dataset (*q*) were estimated based on the *C. dubius* deme, so individuals with high *q* had high *C. dubius* ancestry, and individuals with low *q* had high *C. boraceiensis* ancestry. Therefore, *q* values indicate proportions of an individual's genome attributed to each source population (Shastry et al., 2021). Additionally, we estimated clinal transitions of dorsal morphology based on the proportion of individuals with the uniform dorsal morphology typical of *C. dubius*. We fit clines with a null model and 15 cline models based on all possible combinations of trait interval (fixed to 0 and 1, observed values or estimated values) and tail fits (none fitted, left only, right only, mirrored, or both estimated separately). Model selection was performed using corrected Akaike Information Criterion (AICc) scores and the ML model parameters (cline center and width) were extracted from the best model. Coincidence of cline centers and concordance of cline widths were determined using confidence intervals of two log‐likelihood scores.

### 2.8 Ethics statement

This study followed protocols approved by the Ethics Committee on Animal Use of the Universidade Estadual Paulista, Rio Claro, São Paulo, Brazil (CEUA #038/2015). Field research, collections, and export permits were authorized by the appropriate Brazilian agencies (COTEC‐IF #350/2016 and ICMBio #47515–5). Our data were registered with the appropriate Brazilian agency (SISGEN ADE8BEB) for required documentation of genetic patrimony.

## 3 RESULTS

### 3.1 Mitochondrial haplotype network

**FIGURE 2:**
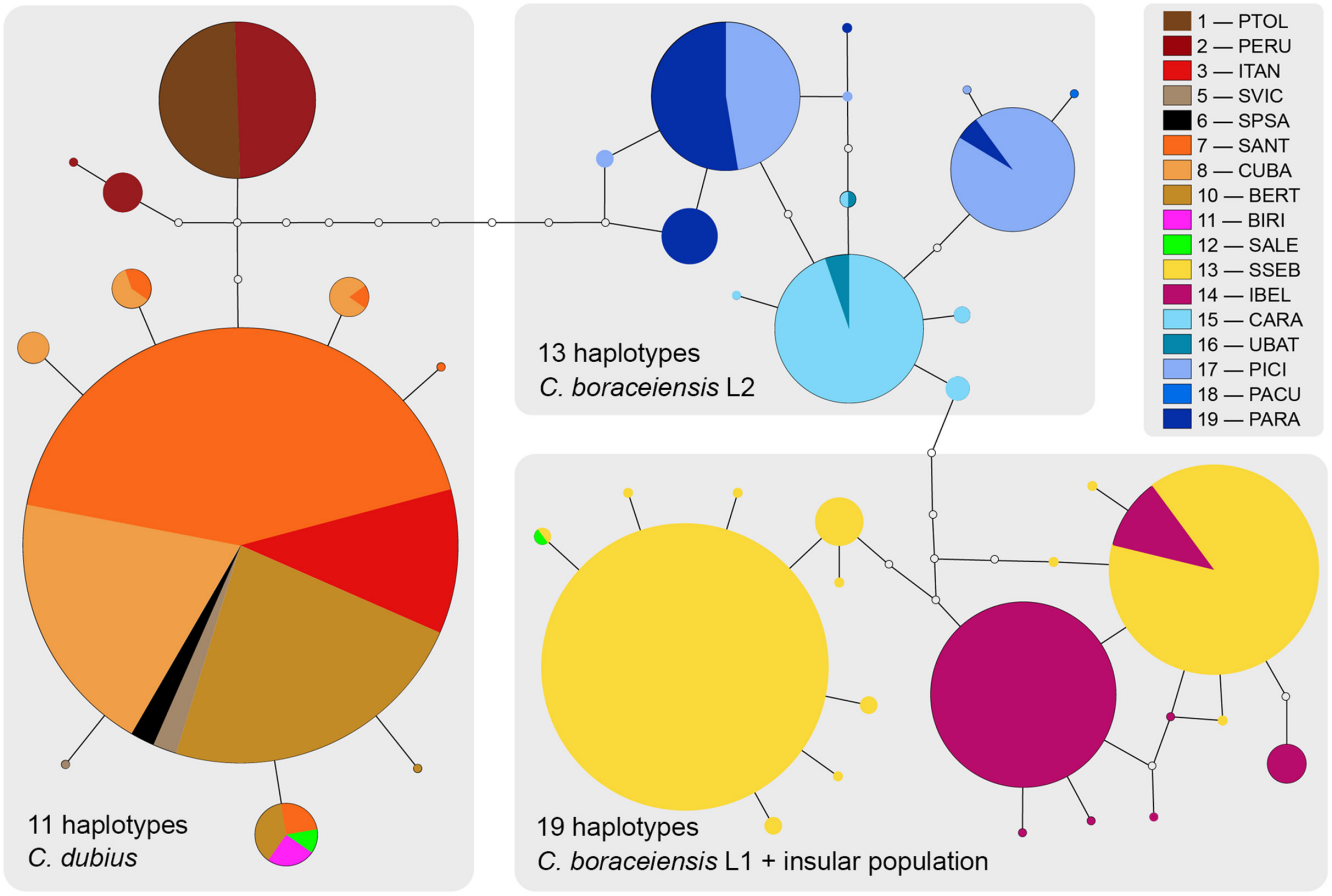
TCS haplotype network based on the alignment of 615 bps of the mitochondrial gene 16S, showing the relationships among all haplotypes of *Cycloramphus dubius* and *Cycloramphus boraceiensis* (*C. boraceiensis* L1 + insular population and *C. boraceiensis* L2). Open circles correspond to missing (or hypothetical) haplotypes and each line between two haplotypes represents one mutational step. Circle sizes depict frequency of haplotypes. The three main haplogroups are encompassed by the light gray boxes. Numbered populations are defined in Table 1.

We identified a total of 43 mtDNA haplotypes, including 11 haplotypes of the *C. dubius* lineage and 32 of two *C. boraceiensis* lineages (Figure 2). Haplotypes of *C. dubius* and *C. boraceiensis* differed by a minimum of nine mutational steps. The 11 haplotypes of *C. dubius* are present at sites 1–12. The 32 haplotypes of *C. boraceiensis* consisted of 19 haplotypes present at sites 12–14 (hereafter called lineage *Cycloramphus boraceiensis* L1, which includes the insular population) that differed by a minimum of five mutational steps from the 13 haplotypes at sites 15–19 (hereafter called lineage *Cycloramphus boraceiensis* L2). The haplotype network shows one of the *C. boraceiensis* L1 haplotypes shared with *C. dubius* at one sampling locality (site 12, SALE). The lineage *C. boraceiensis* L1 is more genetically distinct from *C. dubius* haplotypes than the lineage *C. boraceiensis* L2 (Figure 2); however, *C. boraceiensis* L1 is geographically closer to the contact zone between the two species (Figure 1).

### 3.2 Genomic structure and admixture

**FIGURE 3:**
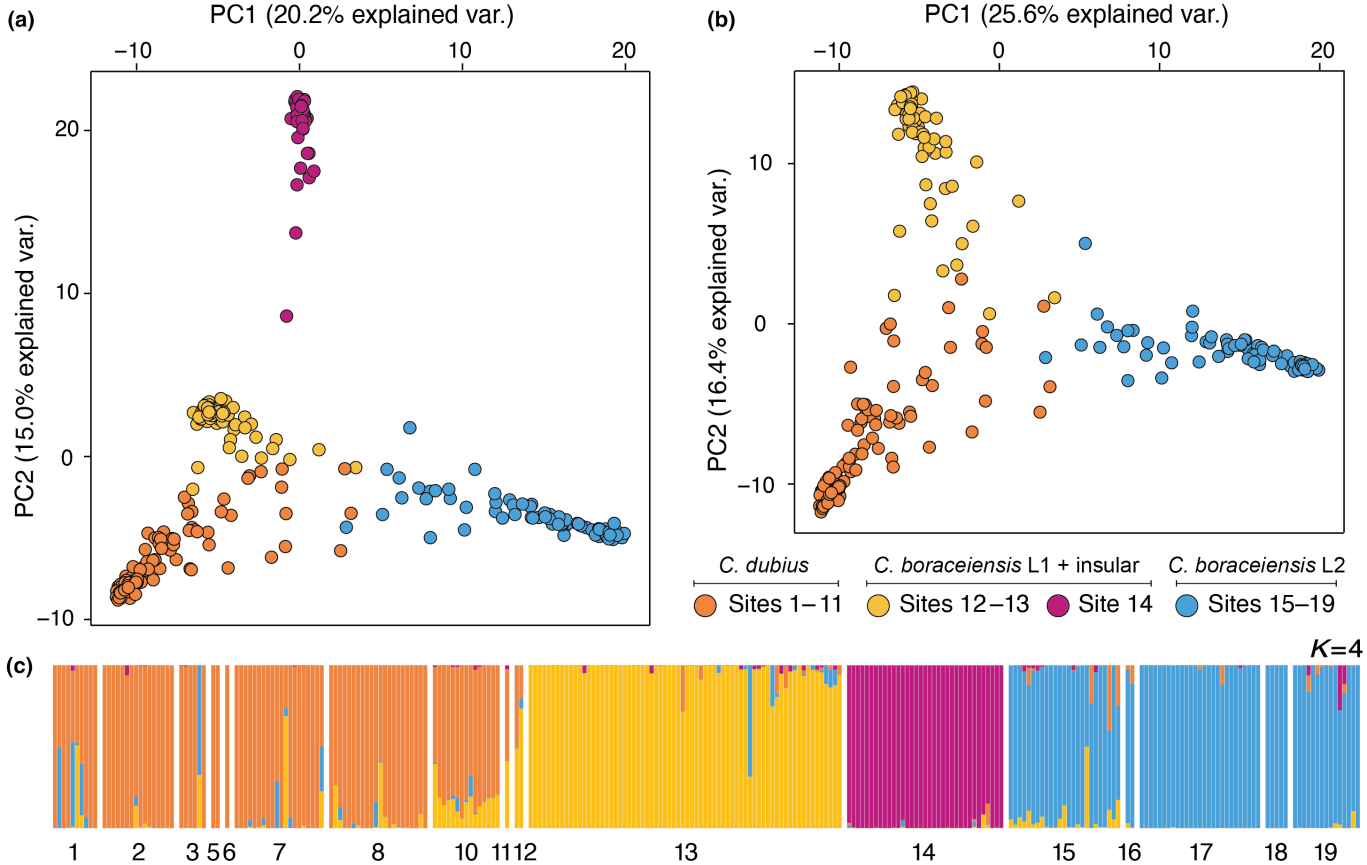
Summary of nuDNA population genetic structure of *Cycloramphus dubius* and *Cycloramphus boraceiensis* (*C. boraceiensis* L1, *C. boraceiensis* insular population, and *C. boraceiensis* L2) based on principal component axis one (PC1) and axis two (PC2) for (a) the full dataset, and (b) dataset with the insular site 14 (IBEL) removed. These axes combined explain over a third of the genotypic variation estimated for 5972 variable nucleotides (SNPs). Points denote individuals and are colored based on majority membership to one of four demes. (c) Bar plot of admixture proportions for *K* = 4. Each bar corresponds to an individual and each colored segment depicts the proportion of an individual genome inherited from one of the inferred source populations. White vertical spaces separate sampling sites. Numbered populations are defined in Table 1.

Our final nuDNA genetic data set included 5972 SNPs with an average of 153× depth of coverage per individual per SNP (±51 SD) and 14.5% missing data. The genetic clustering found in the PCA (Figure 3a) mirrored the geographic distribution of populations (Figure 1). Specifically, PC1, which explained 20.2% of the variance in the data, separated populations from the southwest to the northeast of our sampling range, including a separate cluster of individuals from sites 12 and 13 (SALE and SSEB). Individuals from the isolated insular population (site 14, IBEL) were separated along PC2, which further explained 15% of the variance, and accounted for additional separation of sites 12 and 13 from the remaining clusters. With the isolated insular population removed, the second PCA plot (Figure 3b) clearly showed three clusters with many admixed, intermediate nuDNA genotypes. High levels of population structure and admixture were also evident in the admixture analysis performed in Entropy, indicating that a large fraction of the variation can be accounted for by four ancestral populations (*K* = 4, Figure 3c), corresponding to the four clusters revealed by the PCA analysis. Further partitioning the data into six populations (*K* = 6, Figure S2) revealed additional structure by distinguishing the sampling sites at the extreme ends of our sampling transect. Based on the *K* values evaluated using BIC scores, DAPC identified *K* = 6–10 as the optimal number of clusters found within the data (Figure S3). Additional visual inspection of *K* values ranging from 7 to 8 revealed no additional population clusters, instead highlighting the extent of admixture among populations (Figure S2).

### 3.3 Hybrid ancestry

**FIGURE 4:**
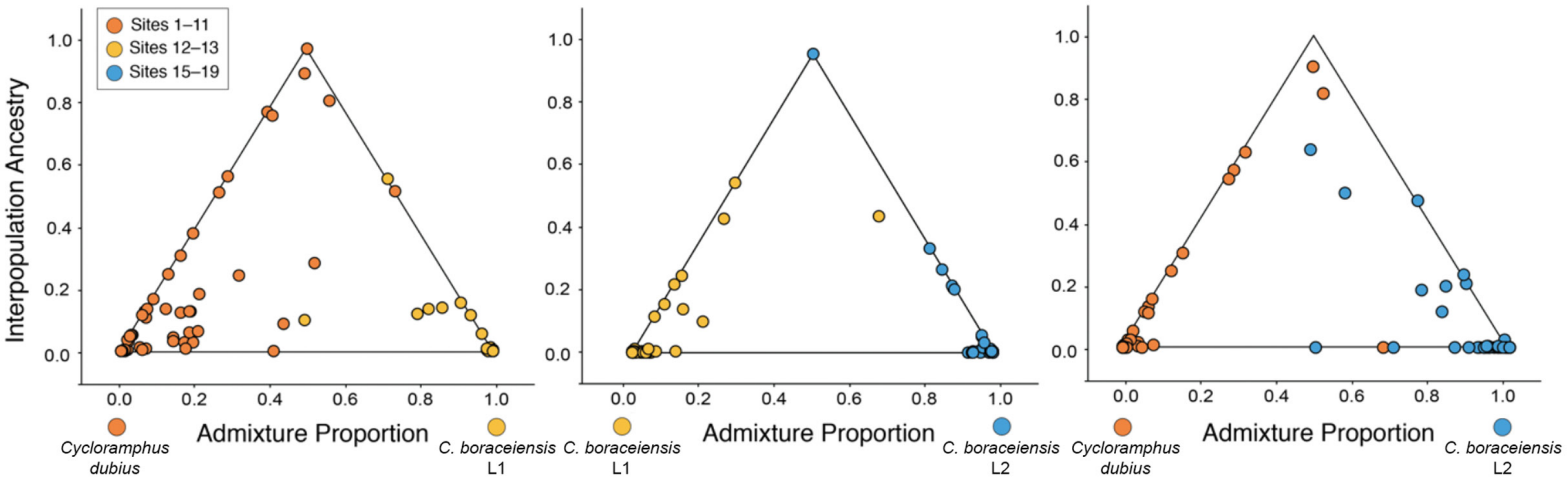
Pairwise hybrid ancestry plots showing the relationship between admixture proportion (*x*‐axes; *q*) and the fraction of loci at which individuals have ancestry from one of two parental populations (*y*‐axes; Q_12_) for *Cycloramphus dubius* and two lineages of *Cycloramphus boraceiensis* (*C. boraceiensis* L1 and *C. boraceiensis* L2). Points denote individuals and are colored based on majority membership to one of three demes. F_1_ hybrids are intermediate in their hybrid ancestry but heterozygous at most of the loci, and therefore fall toward the top of the triangles. Similarly, F_2_ hybrids have a similar genome‐wide level of ancestry, but lower interpopulation ancestry, and fall within the center of the triangle. Backcrossed individuals fall toward the left and right sides of the triangle. Individuals that fall along the *x*‐axis (intermediate admixture proportions, with no interpopulation ancestry) are interpreted as geographic isolation by distance (Gompert & Buerkle, 2016). Numbered populations are defined in Table 1.

We generated three pairwise triangle plots of hybrid ancestry for the three lineages represented among continental populations (Figure 4). We excluded the isolated insular deme on Ilhabela because it showed little evidence of admixture with neighboring demes. Thus, our triangle plots represent hybrids between *C. dubius* (sites 1–11) and *C. boraceiensis* L1 (sites 12 and 13), *C. boraceiensis* L1 (sites 12 and 13) and *C. boraceiensis* L2 (sites 15–19), and *C. dubius* (sites 1–11) and *C. boraceiensis* L2 (sites 15–19). We found evidence of a few F_1_ hybrids between *C. boraceiensis* L1 and *C. boraceiensis* L2, and between *C. boraceiensis* L1 and *C. dubius*. These F_1_ hybrids were intermediate in their hybrid ancestry but heterozygous at most of the loci (points at top of the triangles; Figure 4). We found little evidence of late generation hybrids, which fall within the center of the triangle, having intermediate levels of genome‐wide ancestry but lower interpopulation ancestry (Figure 4). Most hybrids are backcrossed individuals between *C. dubius* and *C. boraceiensis* L1, and between *C. dubius* and *C. boraceiensis* L2, as indicated by the points falling toward the left and right sides of the triangles (Figure 4). We found evidence of more backcrossed hybrids with a pure *C. dubius* parent than any other deme. Finally, individuals that fell along the *x*‐axis (intermediate admixture proportions, with no interpopulation ancestry) are interpreted as showing a pattern of geographic isolation by distance (see Gompert & Buerkle, 2016).

### 3.4 Phylogenetic relationships

The inferred topology from the SNP‐based RAxML‐NG analysis recovers major clades in agreement with our other analyses, with separation between *C. dubius* and *C. boraceiensis*, and population structure within *C. boraceiensis* (*C. boraceiensis* L1, insular *C. boraceiensis*, and *C. boraceiensis* L2). However, the overall topology has relatively low support, especially at the base of the tree (Figure S1). The insular *C. boraceiensis* population is clearly isolated with strong nodal support (site 14, IBEL). Some individuals from site 1 (PTOL; sample 226), site 8 (CUBA; samples 138 and 158), and site 12 (SALE; samples 271 and 273) fall at the base of the *C. dubius* clade and may be the result of admixture or incomplete lineage sorting between *C. dubius* and *C. boraceiensis* L1. Similarly, individuals from site 1 (PTOL; samples 222 and 225) and site 3 (ITAN; sample 268) are potentially admixed between *C. dubius* and *C. boraceiensis* L2, whereas individuals from site 13 (SSEB; sample 201) and site 15 (CARA; sample 248) are potentially admixed between *C. boraceiensis* L1 and L2.

**FIGURE 5:**
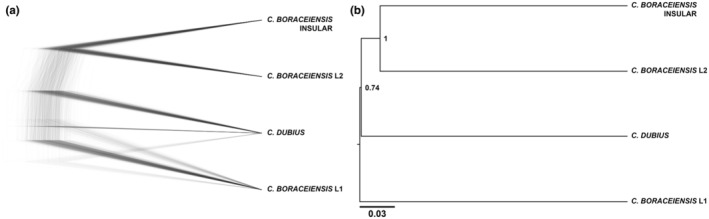
Populations trees for *Cycloramphus dubius* and *Cycloramphus boraceiensis* (*C. boraceiensis* L1, *C. boraceiensis* insular population, and *C. boraceiensis* L2) using a reduced SNP dataset and with only 48 individuals. (a) The posterior distribution of gene trees indicate overall ambiguous relationships among populations, as showed in our cloudogram. (b) SNAPP provides full posterior support only for the insular *C. boraceiensis* and *C. boraceiensis* L2, as showed our MCC tree. Populations are fully defined in Table 1.

Our cloudogram generated via DensiTree analysis, using a reduced SNP dataset and with only 48 individuals, shows the overall disagreement between trees in the SNAPP run. The only two lineages with strong support are the insular *C. boraceiensis* and *C. boraceiensis* L2. All other lineages show topological uncertainty in their relationships. *Cycloramphus dubius*, *C. boraceiensis* L1, and *C. boraceiensis* L2 form a three‐way polytomy at the base of the tree, with some topologies supporting closer relationships between *C. dubius* and *C. boraceiensis* L1 (Figure 5a). This result is confirmed by the MCC analysis (Figure 5b).

### 3.5 Dorsal morphologies and body sizes

Populations varied in the proportions of the three dorsal morphology types (Figure 1). Sites 1–4 exhibited the uniform dorsal morphology typical of *C. dubius* and sites 12, 13, 16–19 exhibited the white‐tubercled dorsal morphology typical of *C. boraceiensis*. Sites 5–8 primarily included the uniform morphology, but also included intermediate morphology at proportions of 9–20%. Sites 9–11 included all three morphologies; site 9 with 80% of individuals exhibiting the uniform morphology and sites 10 and 11 with ≥50% of individuals exhibiting the intermediate morphology. Individuals from sites 14 and 15 mostly exhibit the white‐tubercled dorsal morphology, with a small proportion of intermediate morphology (≤3%). None of the populations exhibited sexual dimorphism in dorsal morphology.

**FIGURE 6:**
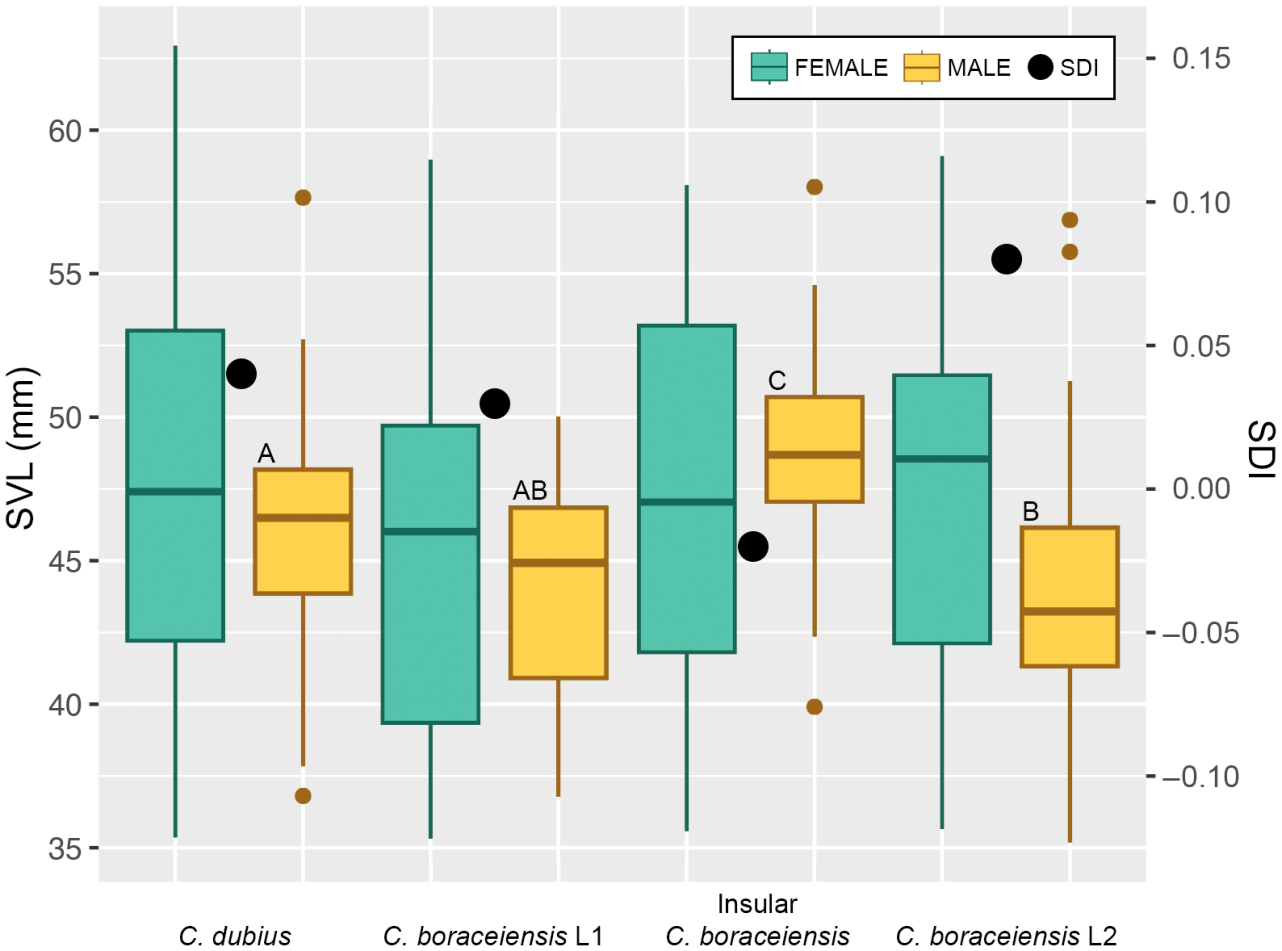
Body sizes by sex and SDIs (=size dimorphism indices; see 2. Materials and Methods for details), all grouped by recovered genetic clusters. *Cycloramphus dubius* occurs from PTOL to BIRI (sites 1–11); southwestern *Cycloramphus boraceiensis* lineage (*C. boraceiensis* L1) occurs in SALE and SSEB (sites 12–13); insular *C. boraceiensis* population occurs in IBEL (site 14); and northeastern *C. boraceiensis* lineage (*C. boraceiensis* L2) occurs from CARA to PARA (sites 15–19). See Figure 1 for site location on map. Numbered populations and sample sizes are fully defined in Table 1. Whereas females are all the same size, males show more variability and statistical differences (different sizes separated by letters A, B, and C on boxes; see results for details).

Females and males were similar in sizes for *C. dubius* (sites 1–11; SDI = 0.04; *t* = 1.82, *p* = .07122), C. *boraceiensis* L1 (sites 12–13; SDI = 0.03; *t* = 0.76, *p* = .45120), and the insular population (site 14; SDI = −0.02; *t* = −0.66, *p* = .51260), but females were significantly larger than males for *C. boraceiensis* L2 (sites 15–19; SDI = 0.08; *t* = 3.89, *p* = .00016). For females, the one‐way ANOVA test showed that all average sizes (grouped by *C. dubius*, *C. boraceiensis* L1, *C. boraceiensis* L2, and the insular population) are not statistically different (*F* = 1.16, *p* = .32574); Tukey test showed that female groups do not vary statistically in body sizes (Table 2 and Figure 6). For males, the one‐way ANOVA test showed that some of average sizes (same grouping as for females) are statistically different (*F* = 9.41, *p* = .00001); Tukey tests showed that males of the insular population are significantly larger than in all other groups, that males of *C. dubius* are significantly larger than males of *C. boraceiensis* L2 but not *C. boraceiensis* L1, and that *C. boraceiensis* L1 and *C. boraceiensis* L2 males do not differ significantly in size (Table 2 and Figure 6). Lastly, focusing particularly on the hybrid zone detected between *C. dubius* and *C. boraceiensis* L1 (sites 1–13), we also grouped adult females and adult males only based on their dorsal morphologies (i.e., independently from their DNA assignments), also excluding intermediate morphologies. We found that females are undifferentiated by their sizes (*t* = 1.84, *p* = .06869), but that males with the typical *C. dubius* dorsal morphology are significantly larger than those with a *C. boraceiensis* morphology (*t* = 2.16, *p* = .03526; Table 2 and Figure 6).

**TABLE 2:** Size variations of adult females and males from each genetic lineage, *Cycloramphus dubius* (sites 1–11), southwestern lineage *Cycloramphus boraceiensis* L1 (sites 12–13), insular *C. boraceiensis* population (site 14), and northeastern lineage *C. boraceiensis* L2 (sites 15–19).

Lineages	Females	Males
Northeastern *Cycloramphus boraceiensis* (=*C. boraceiensis* L2)	47.2 ± 6.01	43.6 ± 4.42
	(35.7–59.1, *n* = 68)	(35.2–56.9, *n* = 64)
Insular *Cycloramphus boraceiensis* (=Ilhabela; IBEL)	47.8 ± 6.98	48.9 ± 4.12
	(35.6–58.1, *n* = 27)	(39.9–58, *n* = 22)
Southwestern *Cycloramphus boraceiensis* (=*C. boraceiensis* L1)	45.2 ± 6.77	44 ± 4.12
	(35.3–59.0, *n* = 31)	(36.8–50, *n* = 21)
*Cycloramphus dubius*	47.6 ± 6.57	45.9 ± 4.04
	(35.4–62.9, *n* = 88)	(36.8–57.7, *n* = 49)
*Cycloramphus boraceiensis* L2 × Insular *C. boraceiensis*	*Q*' = 0.59, *p* = .97510	*Q*' = 7.08, *p* = .00001
*Cycloramphus boraceiensis* L2 × *C. boraceiensis* L1	*Q*' = 2.02, *p* = .48220	*Q*' = 0.53, *p* = .98160
*Cycloramphus boraceiensis* L2 × *C. dubius*	*Q*' = 0.50, *p* = .98530	*Q*' = 3.99, *p* = .02728
Insular *C. boraceiensis* × *C. boraceiensis* L1	*Q*' = 2.18, *p* = .41590	*Q*' = 5.30, *p* = .00145
Insular *C. boraceiensis* × *C. dubius*	*Q*' = 0.25, *p* = .99800	*Q*' = 3.86, *p* = .03514
*Cycloramphus boraceiensis* L1 × *C. dubius*	*Q*' = 2.48, *p* = .29870	*Q*' = 2.39, *p* = .33180
*Cycloramphus boraceiensis* dorsal morphology (hybrid zone)	45.41 ± 6.62	44.19 ± 3.86
	(35.3–59.0, *n* = 34)	(36.8–50.0, *n* = 26)
*Cycloramphus dubius* dorsal morphology (hybrid zone)	47.97 ± 6.64	46.45 ± 3.89
	(35.4–64.9, *n* = 69)	(39.7–57.7, *n* = 29)

*Note*: Values are shown as average (in mm) ±SD; in parentheses range and sample size (*n*). We report *Q*'‐values and *p*‐values from statistical comparisons (Tukey test) of female–female and male–male sizes between different lineages. We also show size variations focusing on the hybrid zone (sites 1–13) but grouping adult females and males by their dorsal morphologies (i.e., independently from their DNA assignments) and also excluding intermediate morphologies. Numbered populations are defined in Table 1.

### 3.6 Cline analyses

**FIGURE 7:**
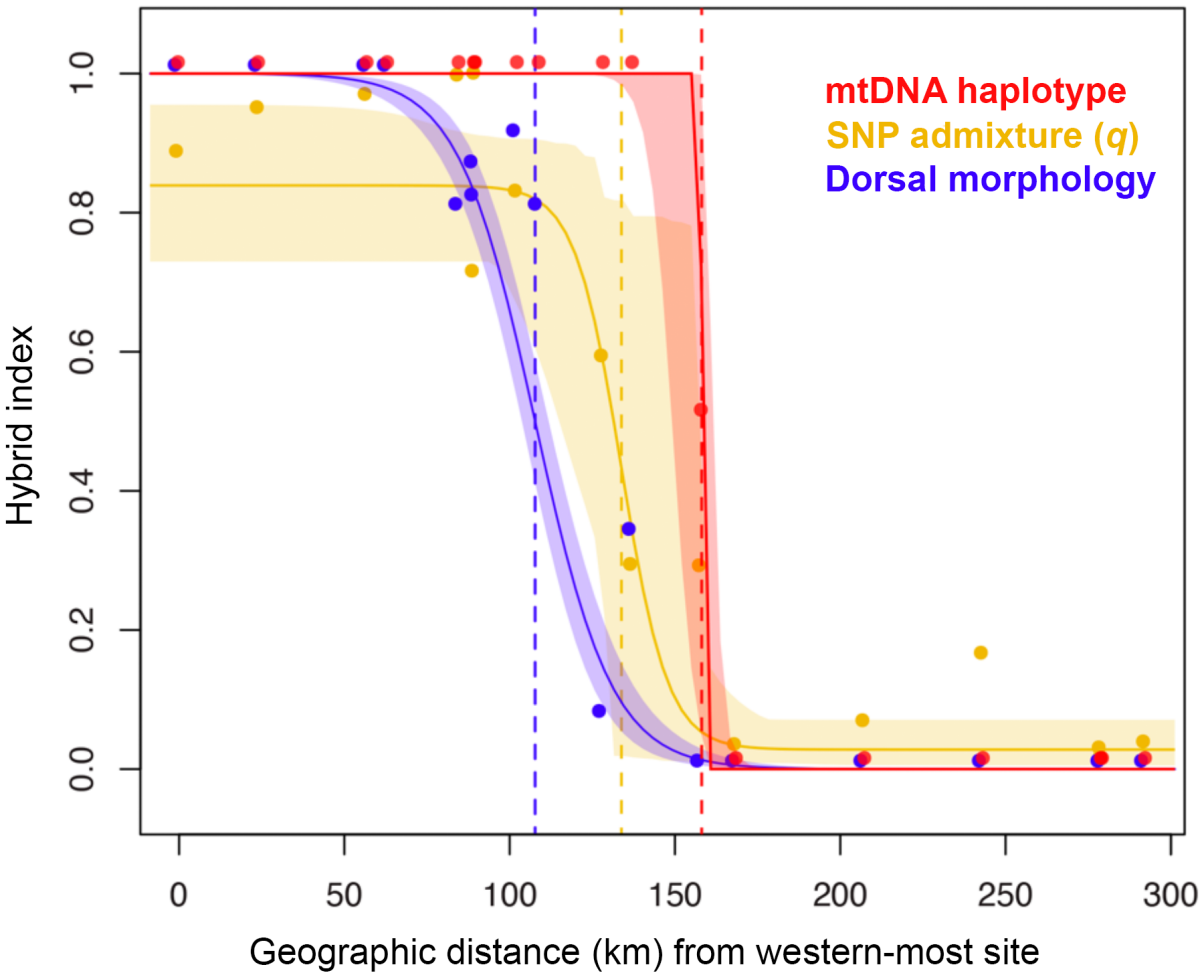
Best‐supported cline models for mitochondrial haplotypes (in red; mtDNA haplotype), genome‐wide admixture proportions based on *K* = 1 (in yellow; SNP admixture, *q*), and (in blue) dorsal morphologies of *Cycloramphus dubius* and *Cycloramphus boraceiensis*. Dotted lines indicate cline centers, and colored shaded areas indicate the confidence intervals.

The best‐supported models included no tail‐fitting for all three estimated clines, fixed trait intervals were selected for dorsal morphology and mitochondrial haplotype frequencies, and a free trait interval based on observed values was selected for *q*. The center of the mitochondrial haplotype cline was estimated near SALE (site 12; Figure 1) at 158.1 km (CI: 147.8–161.8) from the western‐most site, with a width of 0.8 km (CI: 0.2–17.1). For *q*, the cline center was estimated near BIRI (site 11; Figure 1) at 133.8 km (CI: 118.5–145.6) from the western‐most site, with a width of 28.2 km (CI: 0.6–65.6). For dorsal morphology, the cline center was estimated farther west at 107.7 km (CI: 103.1–112.4), near ALTO (site 9; Figure 1), with a width of 46.6 km (CI: 38.2–57.2). The log‐likelihood values for the cline centers do not overlap. The center of the mitochondrial cline was estimated near the species boundaries, between BIRI (site 11) and SALE (site 12), whereas the *q* cline center was displaced nearly 24‐km west, indicating mitonuclear discordance in the contact zone between *Cycloramphus boraceiensis* and *C. dubius*. The dorsal morphology cline was displaced yet nearly 26‐km west of the *q* cline center, indicating that the white‐tubercled dorsal morphology typical of *C. boraceiensis* is also discordant from the previous two cline centers (Figure 7).

## 4 DISCUSSION

### 4.1 Diversification across the Atlantic Coastal Forest

Our results provide evidence of phenotypic and genetic variation among populations of *C. dubius* and *C. boraceiensis*, two sister species of frogs endemic to the Atlantic Coastal Forest in southeastern Brazil. MtDNA and nuDNA patterns for *C. dubius* are more homogeneous across populations (sites 1–11) and are recovered as a single cluster. Conversely, *C. boraceiensis* populations (sites 12–19) are split along their geographic distribution into three main nuDNA clusters: southwestern (lineage *C. boraceiensis* L1), insular (Ilhabela; site 14, IBEL), and northeastern (lineage *C. boraceiensis* L2) lineages. The four recovered clusters are further supported by the ddRADseq‐based PCAs and the phylogenetic analyses, but with important evidence of introgression among lineages. The insular mtDNA haplotypes from Ilhabela (site 14) fall within the southwestern cluster (*C. boraceiensis* L1 haplogroup) with some haplotype sharing with coastal populations. In contrast, the nuDNA data show that admixture between insular and *C. boraceiensis* L1 populations is minor. This pattern of insular genetic differentiation is typical of a recent separation, likely with insular *C. boraceiensis* populations isolated by sea‐level oscillations during the late Pleistocene and Holocene (Grazziotin et al., 2006; Marques et al., 2002; Sabbag et al., 2018; Suguio & Martin, 1978). Commonly, when mainland populations have geographic structure and are large, and/or when island isolation happened recently, the small population isolated on the island renders the mainland populations paraphyletic (Avise, 2000; Funk & Omland, 2003; Johnson et al., 2000; Moritz, 1994). Most continental islands off the Atlantic Coastal Forest were connected to the mainland for the last time during the last glacial maximum, ~13 thousand years ago (Fleming et al., 1998; Souza et al., 2005; Suguio & Martin, 1978). Over multiple periods of sea‐level oscillations and marine introgressions, these islands became fully isolated during the Holocene, when the relative sea level rose from approximately −60 m to levels that we observe today (Fleming et al., 1998; Souza et al., 2005; Suguio & Martin, 1978). These sea level fluctuations promoted different levels of isolation across taxa, with some organisms reaching speciation via vicariance (Brasileiro et al., 2007; Grazziotin et al., 2006; Marques et al., 2002).

Geotectonic structures might explain phylogeographic breaks commonly observed in the Atlantic Forest (Amaral et al., 2013; Amaro et al., 2012). Overall, the distributions of *C. dubius* and *C. boraceiensis* along the Brazilian Atlantic Coastal Forest are associated with escarpments of the Serra do Mar Mountain chain, and both species have their geographic limits that closely match hydrographic basins of the Brazilian coast, with *C. dubius* located in the Santos Lowlands basin and *C. boraceiensis* in the North Coast basin. The topography of the Serra do Mar might also explain diversification within *C. boraceiensis*, because Serra do Mar runs parallel to the Brazilian coastline, including high escarpments closer to the ocean near the city of São Sebastião, São Paulo state (site 13, SSEB; Campanha et al., 1994), possibly acting as natural barriers to gene flow between southwestern and northeastern *C. boraceiensis* lineages (*C. boraceiensis* L1 and *C. boraceiensis* L2). Previous research in this biome has revealed that diversification within the Atlantic Forest is attributable to several mechanisms. Most likely, different historical evolutionary processes occurred throughout the history of the Atlantic Forest and different endemic organisms have responded differently to these processes, due to their ecological requirements (Batalha‐Filho et al., 2012; Condez et al., 2016; Paz et al., 2020; Santos et al., 2020). The split observed between *C. boraceiensis* L1 and *C. boraceiensis* L2 is geographically coincident with lineage divergences in other organisms. Populations of the cycloramphid frog *Thoropa taophora*, which is in the same family as *Cycloramphus*, also show a split in the region of São Sebastião (Duryea et al., 2015; Sabbag et al., 2018). Likewise, the terrestrially breeding *Ischnocnema parva* frog complex from the family Brachycephalidae (Gehara et al., 2017) and the aquatically breeding *Paratelmatobius poecilogaster* frog complex from the family Leptodactylidae (Santos et al., 2020) have lineages splitting around São Sebastião. As we accumulate evidence from phylogeographic datasets for different taxa, we will illuminate general mechanisms leading to the diversification in this biodiversity hotspot.

### 4.2 Taxonomic remarks

Our two focal species have high levels of similarity in morphology, behaviors, and ecological niches, and are also closely related (de Sá, Haddad et al., 2020; Giaretta & Cardoso, 1995; Giaretta & Facure, 2003; Heyer, 1983; Pedrozo et al., 2024). Nonetheless, they are phenotypically distinguishable (Heyer, 1983) and our mitochondrial data confirm that they belong to independent haplogroups, even with partial sympatry. Heyer (1983) wrote that “*C. boraceiensis* most closely resembles *C. dubius*”, but also indicated that *C. dubius* lacks distinct dorsal, white‐tipped tubercles. We verified this taxonomic diagnostic characters by examining the dorsal morphology of seven *C. dubius* and 29 *C. boraceiensis* individuals from the type series (housed at the MZUSP) as well as samples from outside the contact zone, and concluded that the two species are in fact diagnosable throughout much of their ranges based on dorsal tubercles, but in addition they also have differences in other phenotypic characters, with *C. dubius* showing a more granular dorsal texture and with *C. boraceiensis* showing slightly more developed foot webbing. The designated type locality for *C. dubius* is Alto da Serra, Paranapiacaba, Santo André, São Paulo, and the type locality for *C. boraceiensis* is Estação Biológica de Boracéia, Salesópolis, São Paulo (Frost, 2023; Heyer, 1983), and the holotypes show the set of diagnostic phenotypes for each species. Our results uncover an interesting taxonomic challenge, because both type localities fall within the hybrid zone we characterized in this study. We did not have access to DNA samples from the type locality of *C. dubius* and the two individuals of *C. boraceiensis* we had from its type locality were genomically categorized as hybrids. Today, *C. boraceiensis* is extinct at its type locality (Lopes et al., 2020), thus, we do not know if specimens in the type series are hybrids or not. Overall, the fact that our hybrid zone is relatively narrow and that parental forms diagnosed by morphology are found even in populations within the hybrid zone led us to conclude that these two species are maintaining distinct identities, despite genetic introgression. A future re‐evaluation focused on the taxonomy of these sister species is clearly necessary.

In addition to diagnosis of adults, other authors have highlighted the importance of variation in larval stages for *Cycloramphus* taxonomy, based on tadpole phenotypes, development, and behavior (Colaço et al., 2021; Dias et al., 2021; Giaretta & Cardoso, 1995; Heyer, 1983; Heyer et al., 1990; Pedrozo et al., 2024). Also, Carmo et al. (2024) conducted a phylogenetic ancestral reconstruction for the available advertisement call parameters in the genus *Cycloramphus*, and showed that *C. dubius* and *C. boraceiensis* have distinct aspects to their calls, although to date calls have only been recorded from Santos, Salesópolis, and Ubatuba (Giaretta & Cardoso, 1995; Heyer, 1983; Heyer et al., 1990; Heyer & Mello, 1979; Pedrozo et al., 2024). Further studies of variation in these other potentially diagnostic larval and behavioral traits would greatly add to our taxonomic evaluation.

### 4.3 Insights on evolutionary mechanisms operating in this discordant hybrid zone

We examined variation in dorsal morphology for *C. dubius* and *C. boraceiensis*, and detected more diversity than has been previously described (Heyer, 1983). Except for a few individuals with intermediate morphologies, most individuals in the three *C. boraceiensis* genetic clusters are monomorphic (white‐tubercled). The single *C. dubius* genetic cluster is highly polymorphic, with individuals expressing the three dorsal morphologies: uniform at sites 1–11, intermediate at sites 5–11, and white‐tubercled at sites 9–12. It is unknown if the diversity detected in dorsal morphologies is associated with any sexual selection mechanism, but it could be the result of expressed species‐specific phenotypes, with intermediate morphologies indicating mostly F_1_ and backcrossed hybrids.

As with the dorsal morphology, we identified a well‐defined northeastern–southwestern nuDNA cline between *C. dubius* and *C. boraceiensis*, with introgression and hybrid individuals (sites 9–12). The nuDNA‐based PCAs and hybrid ancestry plots further confirm introgression between the two species. In addition, Salesópolis (site 12, SALE) is the only sampled population with *C. dubius* and *C. boraceiensis* (*C. boraceiensis* L1) individuals sharing mitochondrial haplotypes. Combined, these data confirm the existence of an asymmetric hybrid zone, showing mitonuclear and phenotypic discordances. There is evidence of introgression between the northeastern *C. boraceiensis* genetic cluster (*C. boraceiensis* L2) and *C. dubius* populations, even though these clusters currently have disjunct geographic distributions (i.e., allopatric populations), likely indicating retention of ancestral polymorphism or an old contact that is today inexistent. In contrast, the introgression between the southwestern *C. boraceiensis* genetic cluster (*C. boraceiensis* L1) and *C. dubius* populations is ongoing and today these clusters partially overlap. *Cycloramphus dubius* populations show introgression in dorsal morphology and nuDNA variation, but little mitochondrial diversity. Differences in the adaptive landscapes for nuDNA and mtDNA and/or demographic asymmetries determine spatial patterns of mitonuclear discordance; female‐biased dispersal or disparities in female abundance in mixed populations are some factors that alter encounter probabilities and facilitate differential introgression of mtDNA and nuDNA (Funk & Omland, 2003). Hybridization is not uncommon among closely related frog species and has been observed even in species that show elaborate courtship behaviors or variation in sexually selected phenotypic traits, conditions that likely mediate assortative mating (Akopyan et al., 2020; Nali et al., 2022). In the *Cycloramphus* hybrid zone described here, populations of the two species do not appear to face geographic, ecological, behavioral, or biological barriers to reproduction. Our findings suggest that pre‐ or post‐zygotic reproductive isolation mechanisms are failing to prevent hybridization between these two sister species in secondary contact.

We did detect subtle but statistically significant differences in body sizes between the species. Notably, females are the same size, but males are variable, which may indicate distinct selective forces acting on each sex (de Sá, Haddad et al., 2020). First, larger males found on Ilhabela (site 14, IBEL) might be a consequence of insular effects (Stamps & Buechner, 1985). Second, in the hybrid zone, males of *C. dubius* and males of southwestern *C. boraceiensis* (*C. boraceiensis* L1) are similar in sizes; however, when examining only males with species‐typical dorsal morphologies and grouping these males only by those morphologies, we found that males with typical *C. dubius* dorsal morphologies are significantly larger than those with *C. boraceiensis* dorsal morphologies. We know that both *C. dubius* and *C. boraceiensis* have similar reproductive strategies and behaviors, with males aggressively defending territories that encompass the best egg‐laying sites and guarding their eggs (Giaretta & Cardoso, 1995; Giaretta & Facure, 2003), and with females likely choosing mates based on male traits and territories (de Sá, Haddad et al., 2020; Lipshutz, 2018; O'Brien et al., 2020; Santana et al., 2020; Valencia‐Aguilar et al., 2020). The main hypothesis we posit here is that sexual selection might be an evolutionary factor contributing to the distribution of hybrids in this system. Based on the body size variation of males in the hybrid zone, it is possible that, if any interspecific male–male competition takes place, *C. dubius* would have an advantage, benefiting from their larger size when defending resources and excluding competing males (see de Sá, Consolmagno et al., 2020; de Sá, Haddad et al., 2020; Lipshutz, 2018; Muralidhar et al., 2014; Pearson & Rohwer, 2000), and potentially displacing males of *C. boraceiensis*. Two admixing species differing in body sizes could lead to non‐reciprocal hybridization. Males of the larger species might be chosen by females of the smaller species, but males of the smaller species might be rejected by females of the larger species (Grant & Grant, 1997; Lipshutz, 2018; Wirtz, 1999). Larger males of *C. dubius* might be acting as supernormal stimulus for both conspecific and *C. boraceiensis* females, whereas smaller males of *C. boraceiensis* might be acting as a subnormal stimulus for females (de Sá, Haddad et al., 2020; Lipshutz, 2018; Richardson et al., 2010; Wells, 2010; While et al., 2015; Winkelmann et al., 2014). If females do prefer larger males, female choice would also favor non‐reciprocal introgression.

In anurans, advertisement calls are the primary means by which receivers identify appropriate mates (Hoskin et al., 2005; Jaya et al., 2022; Servedio & Boughman, 2017; Wells, 2010). Frog females can often assess conspecific male traits based on their calls, and use those signals for mate choice (Hoskin et al., 2005; Jaya et al., 2022; Servedio & Boughman, 2017; Wells, 2010). Few advertisement calls have been characterized for *C. dubius* and *C. boraceiensis* across their distributions, but the few calls that have been recorded show some interesting differences. Calls of the two species have a similar range of dominant frequencies, but *C. dubius* males from Santos (site 7; SANT) emit longer calls (0.2 s) than *C. boraceiensis* L1 males from Salesópolis (site 12; SALE) or *C. boraceiensis* L2 from Ubatuba (site 16; UBAT), which emit advertisement calls of 0.03 and 0.06 s in length, respectively (Giaretta & Cardoso, 1995; Heyer, 1983; Heyer et al., 1990; Heyer & Mello, 1979; Pedrozo et al., 2024). We do not know how these bioacoustic differences are interpreted by *Cycloramphus* females, but given the importance of calls in frog female choice, these phenotypes may be important signals involved in hybridization dynamics.

Alternatively, factors other than the male body size or advertisement calls can potentially influence the introgression dynamics detected in this hybrid zone. For example, genetic compatibilities, with females preferring allospecific males because of heterosis benefits or other genetic‐related advantages (e.g., Marr et al., 2002) or chemical communication, with females preferring allospecific males due to chemical signals (e.g., Johansson & Jones, 2007).

Intrasexual competition and differential male fitness is a well‐known selective mechanism in the genus *Cycloramphus* and in other species in the family (de Sá, Consolmagno et al., 2020; de Sá, Haddad et al., 2020; Muralidhar et al., 2014). Our previous work on the genus *Cycloramphus* demonstrated that competition among males is pervasive and an evolutionary driver of male body size (de Sá, Haddad et al. 2020). Here, we propose that male–male competition coupled with female mate choice may also play an important role in shaping the distribution of organellar and genomic variation, and phenotypic variation in a secondary contact zone between two divergent sister species. Future studies examining hybrid zones should also consider the role that sexual selection mechanisms might play in shaping anuran diversification.

## AUTHOR CONTRIBUTIONS

**Fábio P. de Sá:** Conceptualization (lead); data curation (lead); formal analysis (lead); funding acquisition (equal); investigation (lead); methodology (equal); project administration (lead); resources (equal); validation (equal); visualization (equal); writing – original draft (lead). **Maria Akopyan:** Formal analysis (lead); investigation (lead); methodology (equal); visualization (equal); writing – original draft (equal). **Erika M. Santana:** Data curation (equal); investigation (equal); methodology (equal). **Célio F. B. Haddad:** Conceptualization (lead); funding acquisition (lead); investigation (equal); methodology (equal); project administration (lead); resources (lead); software (lead); supervision (equal); validation (equal); visualization (equal). **Kelly R. Zamudio:** Conceptualization (lead); funding acquisition (lead); investigation (lead); methodology (lead); project administration (lead); resources (lead); software (lead); supervision (lead); validation (lead); visualization (lead); writing ‐ original draft (lead).

## FUNDING INFORMATION

This research was supported by grants from São Paulo Research Foundation (FAPESP; grant numbers 2014/24972‐4, 2016/06876‐3, 2018/17993‐6, and 2024/13553‐2 to F.P.S., and 2008/50928‐1, 2014/50342‐8, 2021/10639‐5, and 2024/01541‐0 to C.F.B.H), Coordenação de Aperfeiçoamento de Pessoal de Nível Superior (CAPES – Finance Code 001 to F.P.S), and Conselho Nacional de Desenvolvimento Científico e Tecnológico (CNPq; grant numbers 153753/2024‐2 to F.P.S., 304713/2023‐6 to C.F.B.H., and 401729/2013‐3 to C.F.B.H. and K.Z.).

## CONFLICT OF INTEREST STATEMENT

The authors declare that there are no conflicts of interest.

## BENEFIT SHARING STATEMENT

*Benefits generated*: Benefits from this research accrue from the sharing of our data on public databases as described above.

## Supporting information


Figure S1:

## Data Availability

All mitochondrial, genomic, and phenotypic data generated in this study are available in the text, supporting files, or accessioned in NCBI (PRJNA817554) and Figshare (https://doi.org/10.6084/m9.figshare.19383707; de Sá et al., 2024a, 2024b).
