# Supplementary material for: Mitonuclear and phenotypic discordance in an Atlantic Forest frog hybrid zone
Source: Ecol Evol. 2024 Sep 13;14(9):e70262. doi: 10.1002/ece3.70262 (PMC11393776; doi:10.1002/ece3.70262)
Supplement: Supplementary file 1 — Figure S1: [file ECE3-14-e70262-s001.docx]

**SUPPORTING INFORMATION**

Additional supporting information may be found online in the Supporting Information section.

**
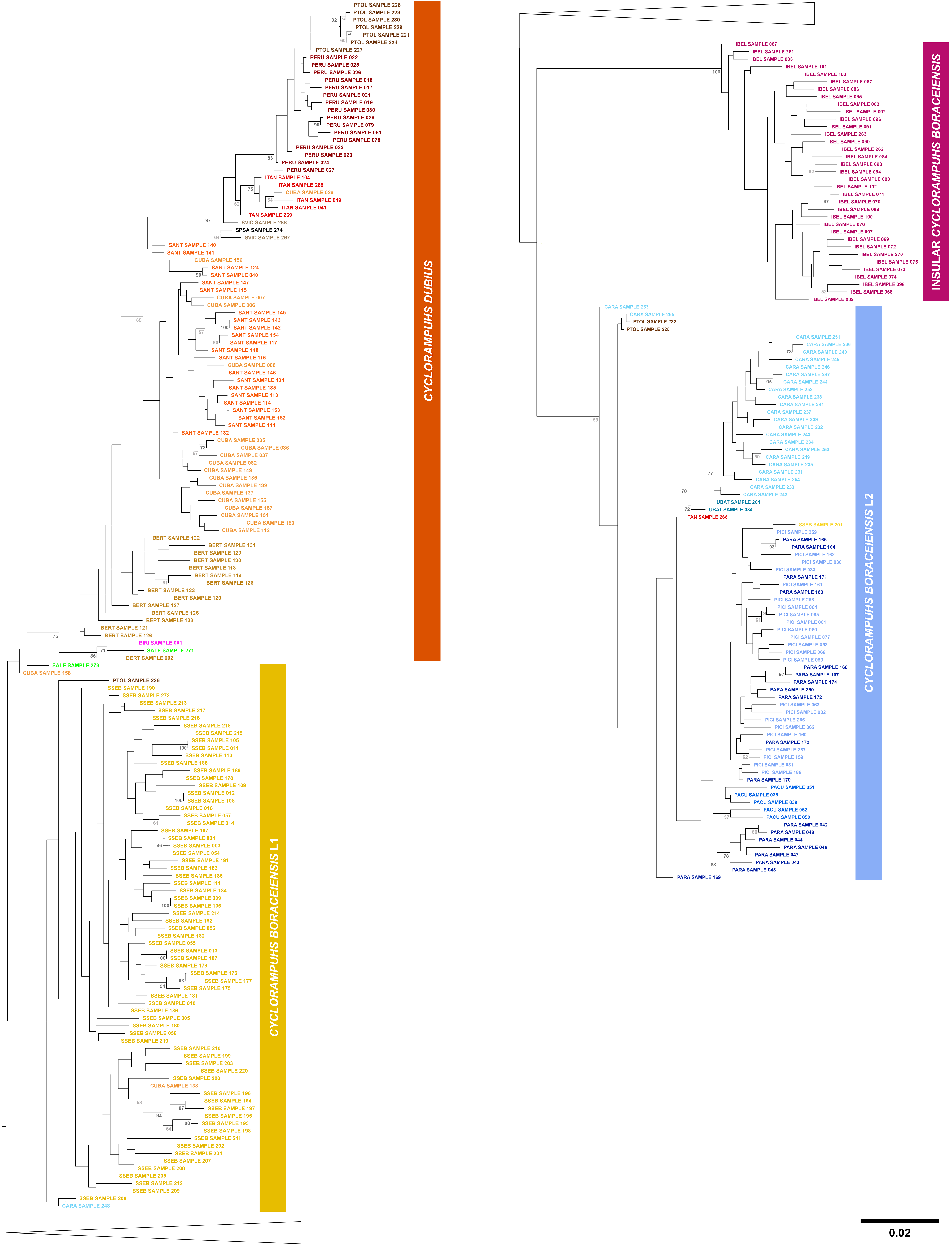
**

**FIGURE S1** Phylogenetic relationships among populations of *Cycloramphus dubius* and *C. boraceiensis*, inferred by maximum likelihood analysis and using genome-wide SNPs. Populations are fully defined in Table 1. Node numbers in the midpoint-rooted tree are bootstrap support values (only values > 50%).


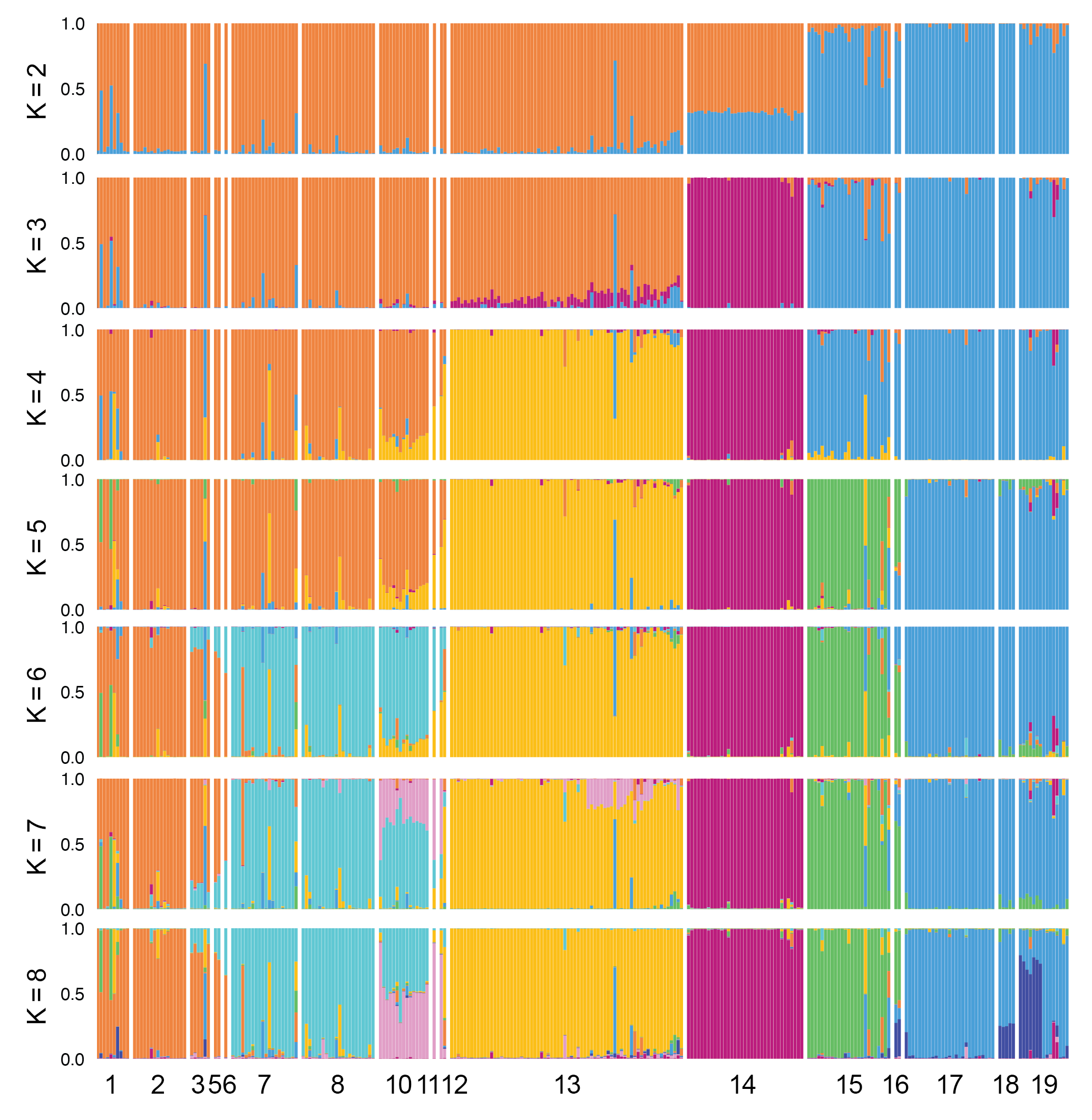


**FIGURE S2** Plots of admixture proportions for K = 2 through K = 8 based on single nucleotide polymorphism (SNP) data. Each bar depicts an individual, each colored segment depicts the proportion of an individual genome inherited from one of the inferred populations. White vertical spaces separate sampling localities. Populations are ordered along the X-axis from 1–19. Numbered populations are defined in Table 1.


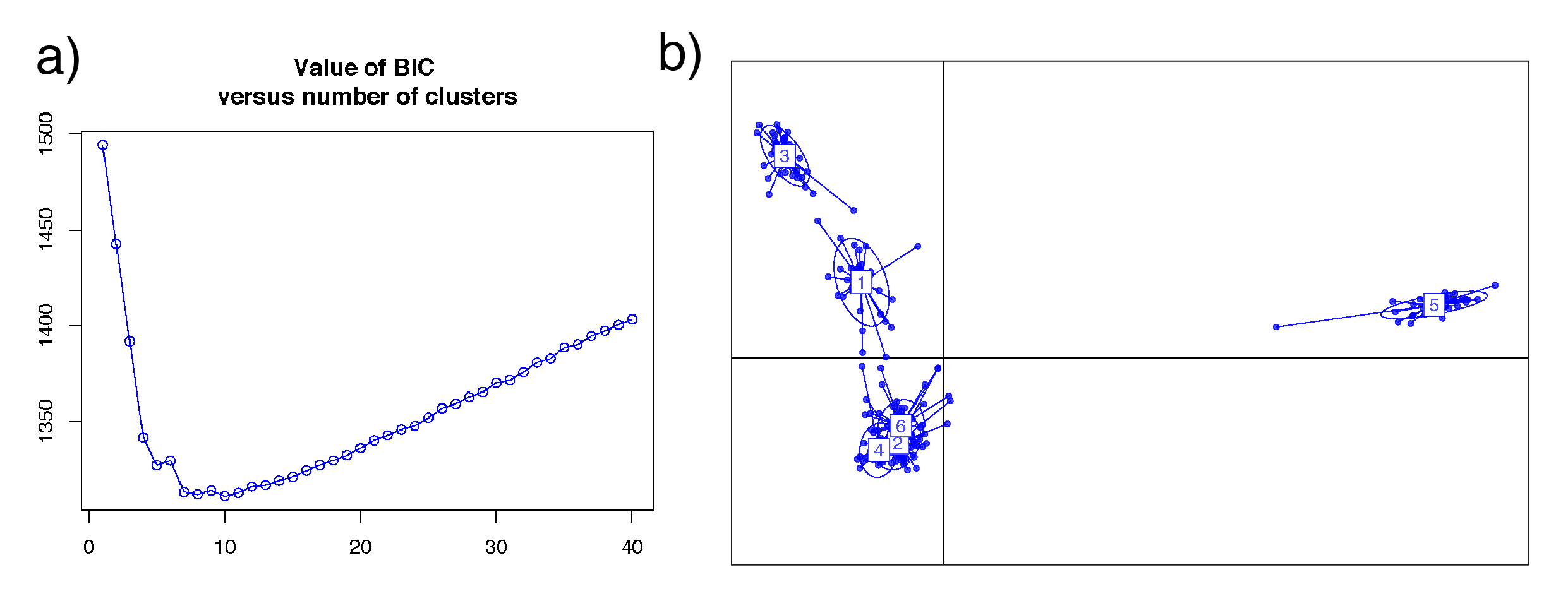


**FIGURE S3** DAPC results showing (a) support for K = 6–10 and (b) plot of six clusters.
